# Living arrangements, health outcomes, and the buffering role of social capital among older adults in China

**DOI:** 10.3389/fpubh.2025.1469914

**Published:** 2025-03-10

**Authors:** Jichao Zheng, Zeqiang Ni

**Affiliations:** ^1^Department of Economics Research, Anhui Academy of Social Sciences, Hefei, China; ^2^School of Economics and Management, Hefei University, Hefei, China

**Keywords:** living arrangements, older adults, health outcomes, social capital, buffering role

## Abstract

**Background:**

Currently, about 40 million older people in China live alone or in nursing homes, and this number is expected to continue increasing. It is important to examine the effects of living arrangements on older people’s health status and determine whether different types of social capital help buffer the health effects of living alone or in nursing homes.

**Method:**

We used data from the CLHLS survey (2017–2018), which included 9,669 older adults. Among them, 1,542 lived alone, 312 lived in nursing homes, and 7,815 lived with their family. We used linear regression to examine the relationship between living arrangements, health outcomes, and social capital.

**Results:**

Living alone leads to higher levels of depression in older adults, with a coefficient of 1.63 for the depression value for older adults, and living alone reduces older adults’ self-rated health scores, with a coefficient of −0.12 on the self-rated health scores for older adults. Living in nursing homes also has a significant effect on the mental health of older adults, with a coefficient of 1.31 for the depression value for older adults. At the same time, we find that different categories of social capital buffer the impact of living alone and living in nursing homes on health. We find that visits from children helped mitigate the increase in depression associated with living in nursing homes and the decrease in self-rated health associated with living alone. At the same time, social interactions at the community level help mitigate the increase in depression values and the reduction of self-rated health scores associated with living alone.

**Conclusion:**

Different categories of social capital buffer the health of older people living alone and in nursing homes differently, so targeted enhancement of social capital based on older adults’ living arrangements is essential to improve their health.

## 1 Introduction

By 2022, the number of people over 65 in China has reached 209.78 million, accounting for 14.9% of the total population (1). Traditional Confucian doctrine emphasizes the filial piety of children, especially sons, to their parents (2). Therefore, in the past, older people in China usually lived with their adult sons. However, with the changes in Chinese society, the traditional living arrangements of older adults have changed, more and more older people are living alone or in nursing homes because they have no children or their adult children are working far from home. According to the seventh population census in 2020, there were 29.94 million households of older persons living alone in the country (3). At the same time, the data show that the total number of beds in China’s long-term care institutions was 8.294 million in 2022 (4).

A large body of evidence suggests that living arrangements have a significant impact on the health status of older people, particularly in terms of depression and self-rated health (5). Since the total number of older adults living alone and in nursing homes in China is close to 40 million, the well-being of this large group deserves attention. This demographic reality highlights the need to study the complex relationship between living arrangements and the determinants of health. Social capital, defined as the sum of the resources embedded in the network of relationships that an individual possesses (6), has been demonstrated to have a significant impact on an individual’s health status (7, 8). Who a person lives with affects their daily socialization patterns and available resources (9). Specific living arrangements—particularly living alone or in nursing homes—may substantially modify social capital through altered family relationships and community engagement patterns. Therefore, exploring the role of this social capital change on the health of older people living alone and in nursing homes makes sense. This paper provides an in-depth study of social capital, living arrangements, and their effect on the health of Chinese older adults.

## 2 Literature review

### 2.1 Pathways through which living arrangements influence health outcomes

The term “living arrangements” refers to the structure of one’s family, such as the number of family members and the relationships between them (10). There are several ways in which different living arrangements affect the health of older adults.

First, different living patterns affect the social support available to older persons. Social support is a major contributing factor to the well-being of older people (11). And family relationships provide resources that can help individuals cope with stress and improve well-being (12). As a result of living alone or in nursing homes, older persons lack social support from their families (13, 14). And social support is closely related to health (15).

Secondly, cultural expectations about how to live can also affect the health of older people. Cultural expectations of intergenerational caregiving responsibilities may influence older adults’ expectations of social relationships (16). For example, traditional Chinese filial piety requires adult sons to live with and care for their older parents in extended families (17). As a result, while the majority of older people around them live with their children, those living alone or in nursing homes are often perceived as having a poor parent–child relationship and face psychological stress. An empirical study found that compared with older adults living with their children, living alone was negatively associated with life satisfaction among rural older adults (18).

Thirdly, living alone or in nursing homes directly impacts the social integration of older persons (19). For example, older people who transition into nursing homes need to leave their former familiar environment, adapt to and integrate into a new group, and impress new people, all of which are stressful (20). Those living alone also face difficulties in integrating into society because they lack the social contact that naturally occurs when they live with others (21). For example, a recently widowed man may find himself ill-prepared to maintain his social network in the absence of his wife (22). In short, when a person faces challenges with social integration, they feel socially isolated, and the brain tends to go into self-protection mode, which may lead to health problems (23).

### 2.2 Health effects of living alone

Living alone is associated with worse economic conditions, loneliness, and less social capital. Widows living alone usually have worse economic conditions and are more likely to perceive that their income is inadequate (24). Some studies also suggest that living alone is associated with greater loneliness in men (25). From a social capital perspective, living alone affects older adult’s social capital. Those who live alone have smaller social networks and less access to instrumental and emotional support (26).

As a result, the impact of living alone on health is usually negative; people living alone are at higher risk for a variety of adverse outcomes, especially those related to health (27). One study found that seniors living alone had higher levels of depressive symptoms, and living alone had a greater effect on depression in men than in women (28). Living alone can also affect self-rated health; a study showed that older adults living alone had lower levels of self-rated health than older adults living with others (29). A study in rural India found that older persons who were currently unmarried and living alone were 38% more likely to rate their health as poor than older persons who were currently married and cohabiting (30).

### 2.3 Health effects of living in nursing homes

For seniors, moving into a nursing home means leaving significant others, giving up many social roles, and leaving familiar surroundings and cherished possessions behind (31). Moreover, social contact has become less frequent after living in nursing homes, and it is challenging to have new social interactions while living in an institution (32). Research has found that the transition from a home to an institutional setting may contribute to feelings of loneliness (33). So, social isolation of older people in nursing homes is widespread (34).

Empirical studies have also found that living in nursing homes has a negative impact on the health of older adults. The quality of life of older adults living in nursing homes is significantly lower than that of those living in the community (35). A meta-analysis showed that the overall prevalence of depressive symptoms among nursing home residents in China was 36.8% (36). And one study found that older people living in the community also have better self-rated health than those living in nursing homes (37).

### 2.4 The buffering effect of social capital on health outcomes

Social capital can be broken into the strength of family, relationships with friends and neighbors, religious and community relationships, workplace relationships, civic engagement, and trust (38). Many studies suggest that the higher the social capital, the better the health status. For example, during the COVID-19 pandemic, when people have positive expectations about the trustworthiness of others and governments, their pandemic-related stress and anxiety would be reduced (39).

Because social capital is associated with health, some scholars believe that social capital is a protective health factor (40). The social capital buffer theory explains the protective role of social capital on health. Social capital can affect health through two mechanisms: first, social support can mitigate the impact of stress assessments by providing solutions to problems and reducing people’s perception of the importance of the issues (41); the second mechanism is that perceived differences in social capital can impact mental health, and the belief that others will provide necessary resources can dampen emotional and physiological responses to stressful events (42). In addition, social capital has a protective effect on people with lower levels of education; some studies have concluded that social capital has greater positive health effects on those with low personal capital than those with high personal capital (43). Several studies have confirmed the buffering effect of social capital between living arrangements and health. A study found that social support is a strong mediator of the impact of living arrangements on mental health (44). Moreover, older adults with good social networks have higher levels of mental health regardless of their living arrangements (45).

After combining existing research, we find that different living arrangements affect older people’s health status and social capital, and social capital has a buffering effect on unfavorable living arrangements. It is natural to speculate whether the negative health effects of living alone or in nursing homes can be buffered by filling the social capital deficit caused by changes in living arrangements. Clarifying this buffering effect will help us formulate social capital generating policies to improve the health of older adults living alone or in nursing homes. Currently, there is a lack of research using Chinese data to study the health of institutionalized older adults, and even less literature on the buffering effect of social capital on the health of older adults living alone and in institutions. This paper aims to contribute to research in this area by utilizing Chinese data to examine the effects of living alone or in nursing homes on the health of older adults and to further explore the role of social capital in buffering the impact of living arrangements on health.

## 3 Methodology and design

### 3.1 Data sources

This study used data from the Chinese Longitudinal Healthy Longevity Survey (CLHLS) survey (2017–2018). The CLHLS is a tracking survey of older adults organized by Peking University and the National Development Research Institute, which covers most of China’s provinces, cities, and autonomous regions. The questionnaires cover the basic conditions of older adults and their families, socio-economic background and family structure, economic sources, self-rated health and quality of life, personality and psychological characteristics, ability to carry out daily activities, lifestyle, etc. The most recent tracking survey (2017–2018) interviewed 15,874 older people, and the CLHLS provides data free of charge to scholars (46, 47). The current retirement age in China is 60, and the social customs also generally recognize that people over 60 can be considered older adults. In line with the purpose of the study, we restricted the study sample to older people over the age of 60 who had no missing values in any of the selected variables.

### 3.2 Dependent variables

Health variables include depression and self-rated health. Depression is a common mental health problem and one of the major global disease burdens (48). Many studies focusing on mental health use depression as a core indicator (49). Self-rated health is recognized as a subjective measure that integrates an individual’s physical, psychological, social, and functional aspects; it has been widely accepted as a reliable indicator of overall health (50).

The depression variable in the CLHLS survey was measured using a simplified version of the CESD scale with 10 questions. The 10-item scale was found to have adequate reliability and validity (51). The 10-item scale consists of seven negatively scored questions, such as “Are you bothered by things that do not usually bother you?” and three positively scored questions, such as “Do you feel hopeful about the future?” Each question had five answers: always, often, sometimes, seldom, and never; each answer was coded 1, 2, 3, 4, or 5 for the positively scored questions and 5, 4, 3, 2, or 1 for the negatively scored questions. We summarized the answers to the 10 questions and constructed a continuous variable ranging from 10 to 50, with higher values indicating more severe depression.

The self-rated health variable is measured by the question, “How do you rate your health at present? “, which was answered with five options: very good, good, so, bad, and very bad; each answer was coded as 5, 4, 3, 2, or 1. Higher values indicate better self-rated health.

### 3.3 Explanatory variables

We focus on two explanatory variables. The first is living arrangements. The questionnaire asked respondents about their living arrangements. There were three types of answers: living with family, living alone, and living in a nursing home. Based on the answers to the above question, we categorized each respondent’s living arrangement into living with family, living alone, and living in a nursing home. We use three dummy variables to represent each of these three states.

Social capital is another explanatory variable we focus on. According to the social–emotional choice theory, in old age, when time is perceived to be limited, people value short-term goals such as emotional regulation more than long-term goals such as access to information (52). In practice, it has been found that, as they age, older people place more emphasis on interactions with close relatives in the composition of their social networks (53). This means that interactions with their children are at the core of older people’s social interaction activities. Neighbors and friends are also important in social networks (54). Based on the above theoretical analysis, we used structural social capital, i.e., family- and community-level social capital.

Family-level social capital refers to children’s visits to the respondent. The questionnaire asks, “Do your children visit you often?” One child who visits the respondent regularly is assigned a value of 1, two children who visit regularly are assigned a value of 2, and so on. The larger the variable’s value, the more often the children see the respondent and the more help they provide to the respondent.

Some studies have identified neighborhood interactions are an important component of social capital at the community level (55). We used social interactions as community-level social capital, with the questionnaire asking, “Do you now perform the following activities regularly?” We chose two types of social activities to represent community-level social capital: visiting and interacting with friends, and playing cards or mah-jong. The answers to these questions are: almost every day; not every day, but at least once a week; not every week, but at least once a month; not every month, but sometimes; never. We assigned values from 4 to 0 to each answer, respectively. We summed the answers to the two questions above to obtain a score of 0–8, with the higher the level of participation in social activities, the higher the value of the variable.

### 3.4 Control variables

The control variables consist of three main dimensions, as shown in Table 1. The first dimension is demographic background factors, including age, gender, marital status, etc. The second dimension is the respondent’s socioeconomic status, measured by several indications: education, pension, sufficient, etc. The third dimension is the respondent’s health status, which mainly includes instrumental activities of daily living, whether or not they smoke, and whether or not they drink alcohol, instrumental activities of daily living.

**Table 1:** Summary of the control variables.

Variables	Original question	Code
Age	Current age.	The variable takes the value of actual age.
Gender	Respondent’s gender.	Male = 1，Female = 0.
Marital status	Current marital status.	Married = 1, Others = 0.
Education	How many years did you attend school?	The variable takes the value of the respondent’s years in school.
Pension	Do you have a pension for retirement?	Have a pension for retirement = 1, others = 0.
Sufficient	Does all of your financial support sufficiently pay your daily costs?	Yes = 1, No = 0.
Smoke	Do you smoke at present?	Yes = 1, No = 0.
Drink	Do you drink alcohol at present?	Yes = 1, No = 0.
Iadl	Can you visit your neighbor’s house, go shopping, cook, wash clothes, walk 1,000 m in a row, lift something weighing about 5 kg, squat down and stand up three times in a row, and travel by public transportation all by yourself? Eight questions in total.	The answers to these questions are: yes, independently; yes, but need some help; no, cannot. We assigned each answer a value of 0 to 2. We summed the answers to the eight questions to obtain a score of 0–16; the higher the score, the worse the ability to carry out daily activities.

### 3.5 Research methodology

We used multivariate regression to examine the effect of living arrangements on the health of older adults. In addition, the interaction terms between living arrangements and social capital were added to the regression equation:


(1) $$\begin{array}{l} \mathrm{Healt}h_{i}=a+\beta_{1}\mathrm{Livingarrangement}s_{i}\\ \;+\beta_{2}\mathrm{SocialCapita}l_{i}+\beta_{3}\mathrm{Livingarrangement}s_{i}\\ \;*\mathrm{SocialCapita}l_{i}+\beta_{n}\mathrm{ControlVariabl}e_{i}+\varepsilon_{i} \end{array}$$

In Equation 1, the Health_i_ value indicates the health status of the respondent_i_. Livingarrangements_i_ is the living arrangements of the respondent. Socialcapital_i_ is the personal social capital variable. Livingarrangements_i_*Socialcapital_i_ is the interaction term.

Where the effect of the living arrangements on health is:


(2) $$\frac{\partial \mathrm{Health}}{\partial \mathrm{Livingarrangements}}=\beta_{1}+\beta_{3}\mathrm{SocialCapital}$$

In Equation 2, *β*_1_ represents the direct effect of living arrangements on health, and *β*_3_ denotes the coefficient of the interaction term. When the coefficient of *β*_3_ is significant, it implies that the social buffers the health shocks generated by living arrangements. Based on this buffering effect, social capital can act as a moderator between living arrangements and health.

## 4 Empirical results

### 4.1 Data description

There were missing values for the variables in our study; for example, 3,365 respondents had missing values for the depression variable, and 1,432 respondents had missing values for the self-rated health variable. After excluding the missing value sample, there were 9,669 respondents. Among them, 1,542 lived alone, 312 lived in nursing homes, and 7,815 lived with their family. The proportions of those living alone and in nursing homes were 15.93 and 3.69%, respectively. Meanwhile, in the total sample, of the 15,548 samples with values for the living arrangements variable, 2,477 lived alone, and 574 lived in nursing homes, for rates of 15.95 and 3.23%, respectively. The distribution of the living arrangements of the sample we used is comparable to that of the total sample, so it can be assumed that there is no distributional bias in our study sample.

The depression levels of different groups are shown in Table 2, which shows that those living in nursing homes had the highest level of depression, with a mean of 23.95; those living with family had the lowest level of depression, with a mean of 21.79; and those living alone had the middle level of depression, with a mean of 23.28. Both living alone and living in nursing homes have a positive impact on the depression value of older adults. Regarding the self-rated health variables, respondents living with their family had the best self-rated health, while those living in nursing homes had the worst.

**Table 2:** Description of variables.

Variables	Full sample	Living with family	Living alone	Living in nursing homes
Depression	22.10301 (6.097754)	21.79655 (6.0006)	23.2821 (6.394062)	23.95192 (5.994177)
Self-rated health	3.472127 (0.894851)	3.478823 (0.895217)	3.457847 (0.899477)	3.375 (0.858334)
Social interactions	2.43324 (2.282268)	2.408573 (2.295555)	2.69131 (2.182025)	1.775641 (2.269818)
Visits	3.118213 (1.800792)	3.119514 (1.76471)	3.231518 (1.934413)	2.525641 (1.899225)
Age	83.15741 (11.34571)	82.76404 (11.67417)	84.22568 (9.610907)	87.73077 (9.443926)
Gender	0.453511 (0.497860)	0.476008 (0.499456)	0.355383 (0.478784)	0.375 (0.484901)
Marital status	0.456407 (0.498122)	0.547793 (0.497743)	0.062257 (0.24170)	0.115385 (0.320)
Education	3.796049 (4.419484)	3.915675 (4.431566)	2.95655 (4.038112)	4.948718 (5.269331)
Pension	0.307684 (0.461559)	0.307614 (0.461535)	0.236057 (0.424795)	0.663462 (0.473285)
Sufficient	0.874754 (0.331014)	0.876008 (0.329594)	0.865110 (0.341717)	0.891026 (0.312108)
Smoke	0.157410 (0.364206)	0.163660 (0.369990)	0.143969 (0.351172)	0.067308 (0.250957)
Drink	0.149136 (0.356241)	0.153935 (0.360909)	0.138132 (0.345151)	0.083333 (0.276830)
Iadl	4.626332 (5.633043)	4.640819 (5.724129)	3.636187 (4.53903)	9.157051 (5.968649)
*N*	9,669	7,815	1,542	312

Data shown are means and standard deviations.

Among other control variables, older adults living in nursing homes had the lowest indices of children’s visits and social interactions, the highest level of education and pension ownership, and the highest IADL values; in general, it can be concluded that older adults living in nursing homes are in poorer health and have the best levels of income, and have less social capital. In contrast, older adults who live alone have the highest indices of children’s visits and social interactions, the lowest education level and pension ownership, and the lowest IADL values. Our study found significant differences in the economic conditions of older adults living alone and those in institutions.

### 4.2 Living arrangements, social capital and depression

We use three models to analyze the linear relationship between living arrangements, social capital, and depression. The explanatory variables in Model 1 include living alone, living in nursing homes, social capital, and control variables. Models 2 and 3 contain interaction terms. Model 2 includes the interaction terms between living alone and social capital, and Model 3 includes the interaction terms between living in nursing homes and social capital. In our study, older people’s living arrangements are represented by three dummy variables, and since living alone and living in a nursing home are unconventional living statuses in China, we conducted regression analyses using living with family as the reference group to analyze the effects of the two statuses, living alone and living in a nursing home, on older people’s health status relative to living with family.

The regression results are presented in Table 3. Model 1 shows that living alone and living in nursing homes have a significant effect on the mental health of older adults, increasing the mean value of depression by 1.63 and 1.31, respectively, which are relatively large values, suggesting that non-normal living arrangements have a negative impact on the mental health of older adults. The “children’s visits” and “social interactions” variables have a significant adverse effect on depression, indicating that the higher the social capital, the better the mental health of older adults. On average, the value of depression decreases by 0.18 for each additional child who regularly visits their parents, and the value of depression decreases by 0.09 for every 1-unit increase in the social interaction index.

**Table 3:** Linear regression of depression on living arrangements and social capital.

Variables	Model 1	Model 2	Model 3
Social interactions	−0.093273^***^ (0.028678)	−0.064914^**^ (0.030864)	−0.092142^***^ (0.029072)
Visits	−0.179974^***^ (0.034578)	−0.158236^***^ (0.038048)	−0.164193^***^ (0.035159)
Living alone (Ref: Living with family)	1.631241^***^ (0.176952)	2.479865^***^ (0.380731)	1.632338^***^ (0.176955)
Living in nursing homes (Ref: Living with family)	1.313532^***^ (0.342991)	1.333474^***^ (0.343103)	2.422894^***^ (0.615648)
Age	−0.036748^***^ (0.007736)	−0.036626^***^ (0.007734)	−0.036809^***^ (0.007738)
Gender	−0.362716^***^ (0.139433)	−0.367661^***^ (0.139407)	−0.364725^***^ (0.139455)
Marital status	0.037222 (0.158112)	0.036027 (0.158553)	−0.042162 (0.158116)
Education	−0.081452^***^ (0.017479)	−0.081407^***^ (0.017475)	−0.081526^***^ (0.017475)
Pension	−0.482705^***^ (0.153627)	−0.473866^***^ (0.153620)	−0.477707^***^ (0.153609)
Sufficient	−3.260904^***^ (0.180349)	−3.259064^***^ (0.180398)	−3.257784^***^ (0.180318)
Smoke	−0.09038 (0.177115)	−0.091054 (0.177067)	−0.086734 (0.177086)
Drink	−0.989394^***^ (0.176213)	−0.984913^***^ (0.176167)	−0.986947^***^ (0.176179)
IADL	0.235645^***^ (0.014796)	0.237046^***^ (0.0148)	0.236151^***^ (0.014799)
Living alone^*^Visits		−0.116218 (0.084172)	
Living alone^*^Social interactions		−0.179359^**^ (0.072897)	
Living in nursing homes^*^Visits			−0.430758^**^ (0.174972)
Living in nursing homes^*^Social interactions			−0.007099 (0.146256)
*N*	9,669	9,669	9,669
Adjusted *R*-squared	0.1104	0.1109	0.1108

^*^p < 0.1; ^**^p < 0.05; ^***^p < 0.01.

Among the control variables, age, gender, education, pension, sufficient, and alcohol consumption were negatively associated with depression values among older adults, whereas the IADL index is positively associated with depression values; the marital status variable and the smoke variable did not have a significant role in influencing depression, which may because most of our sample were older adults, and that the effect of marriage on the mental health of older adults tends to be stabilized.

After adding the interaction term, we find that the adjusted *R*-squared increases for both Model 2 and Model 3, indicating that the explanatory power of the model increases with the addition of the interaction term. The regression results of Model 2 show that the interaction term between living alone and children’s visits is insignificant, suggesting that family-level social capital does not have a buffering effect on the mental health of older adults who live alone. In contrast, the interaction term between living alone and social interactions is significantly negative, indicating that community-level social capital has a strong protective effect on the mental health of older adults who live alone. The regression results of Model 3 show that the interaction term between children’s visits and living in nursing homes is significantly negative, while the interaction term between social interactions variable and living in nursing homes is not significant, suggesting that children’s visits help to buffer the effects on the depression levels of older adults, while the social interactions does not help to buffer the effects of nursing home stay on mental health.

### 4.3 Living arrangements, social capital and self-rated health

We use three models to analyze the linear relationship between living arrangements, social capital, and self-rated health. The explanatory variables in Model 1 include living alone, living in nursing homes, social capital, and control variables. Models 2 and 3 contain interaction terms. Model 2 includes the interaction term between living alone and social capital, and Model 3 contains the interaction term between living in nursing homes and social capital.

The regression results are presented in Table 4. The regression results of model 1 show that living alone significantly impacts the self-rated health of older adults, and living alone decreases the self-rated health index by 0.12, which is a relatively large value. While living in a nursing home has little impact on the self-rated health. Both variables “children’s visits” and “social interactions” have a positive and significant effect on self-rated health, indicating that the higher the social capital, the higher the individual’s self-rated health index. On average, each additional child who regularly visits the respondent increases the self-rated health index by 0.024, and each additional unit of the social interactions index increases the self-rated health index by 0.015.

**Table 4:** Linear regression of self-rated health on living arrangements and social capital.

Variables	Model 1	Model 2	Model 3
Social interactions	0.015018^***^ (0.004256)	0.011619^**^ (0.00458)	0.015077^***^ (0.004316)
Visits	0.023547^***^ (0.005132)	0.016742^***^ (0.005646)	0.022029^***^ (0.005219)
Living alone (Ref: Living with family)	−0.120121^***^ (0.026262)	−0.293892^***^ (0.056493)	−0.120355^***^ (0.026267)
Living in nursing homes (Ref: Living with family)	−0.001005 (0.050905)	−0.006587 (0.050910)	−0.098846 (0.091387)
Age	0.01054^***^ (0.001148)	0.010528^***^ (0.001148)	0.010553^***^ (0.001149)
Gender	0.01336 (0.020694)	0.014467 (0.020686)	0.013424 (0.0207)
Marital status	−0.101915^***^ (0.023466)	−0.104624^***^ (0.023526)	−0.102485^***^ (0.023471)
Education	−0.000673 (0.002594)	−0.000737 (0.002593)	−0.000664 (0.002594)
Pension	0.035410 (0.02280)	0.034329(0.022794)	0.034953 (0.022802)
Sufficient	0.479364^***^ (0.026766)	0.477643^***^ (0.026768)	0.479032^***^ (0.026767)
Smoke	0.006247 (0.026286)	0.006672 (0.026273)	0.00593 (0.026287)
Drink	0.212354^***^ (0.026152)	0.211498^***^ (0.02614)	0.212099^***^ (0.026152)
IADL	−0.039392^***^ (0.002196)	−0.039648^***^ (0.002196)	−0.039453^***^ (0.002197)
Living alone^*^Visit		0.036161^***^ (0.012490)	
Living alone^*^Social interactions		0.0212^*^ (0.010817)	
Living in nursing homes^*^Visit			0.041530 (0.025973)
Living in nursing homes^*^Social interactions			−0.004413 (0.02171)
*N*	9,669	9,669	9,669
Adjusted *R*-square	0.0901	0.0911	0.0902

^*^p < 0.1; ^**^p < 0.05; ^***^p < 0.01.

After adding the interaction term, we find that the adjusted *R*-squared increases for Model 2, indicating that the model’s explanatory power increases with the addition of the interaction term. The regression results of Model 2 show that the interaction terms of living alone with children’s visits and social interactions are both significant, indicating that social capital at the family and community level have a buffering effect on the self-rated health of older adults living alone. The regression results of Model 3 show that both interaction terms between living in nursing homes and social capital are insignificant, indicating that frequent visits from children and community-level social interactions do not help to buffer the adverse effects of living in nursing homes on self-rated health.

## 5 Discussion and recommendations

### 5.1 Discussion

Our study shows that living alone or in a nursing home has a detrimental effect on the physical and mental health of Chinese older adults; at the same time, our study finds that social interactions are very beneficial to the physical and mental health of older adults living alone, and that visits from children can help to improve the mental health of older adults living in nursing homes, as detailed in the following results. Our findings have important implications for the improvement of the health of Chinese older adults.

First, our findings suggest that older Chinese adults who do not live with their families have poorer health statuses, possibly because traditional Chinese culture emphasizes filial piety, which means that adult sons should live with and care for older adults. This differs from other developed countries; for example, the Nordic countries have the highest proportion of older persons living alone, ranging from 45% to 50% for women and nearly 25% for men (56). Living alone and living in nursing homes are non-traditional living patterns; the most common reason for living alone is widowhood, and because social customs do not encourage remarriage for older adults, these widowed older adults have to live alone, and they often have worse mental health (57). In addition, there is also a stigma attached to living in nursing homes, as traditionally, only “widows and widowers” without sons are admitted to nursing homes. Meanwhile, rumors of “nursing home abuse” often appear in the social news. Nursing home abuse is widespread, with the prevalence rates in Macao and Guangzhou standing at 11.48% and 8.24%, respectively (58). Therefore, these two living arrangements hurt older adults’ health.

Second, social interactions have a more significant buffering effect on the health status of older adults living alone. For older adults living alone, both family-level social capital and community-level social interactions buffer the adverse effects of living alone on self-rated health. Meanwhile, social interactions can help to buffer the adverse effects of living alone on depression. This may be because older adults who live alone are generally in good health; they still can maintain social relationships. Frequent visits from children may help to improve their self-rated health, but socializing with one’s peers is more likely to alleviate feelings of loneliness and reduce the adverse health effects of living alone. This conclusion is consistent with many studies. For example, a study in the United States found that interventions to promote social participation can improve older people’s physical and mental health (59). One other study also found that social interactions can buffer the effects of widowhood on functional ability (60).

Third, social capital at the family level has a more significant buffering effect on the mental health of older adults living in nursing homes. Older adults living in nursing homes tend to be older and less able to care for themselves, making it difficult for them to go out for social interaction at the community level. Instead, according to the socioemotional selectivity theory, they will pursue emotionally meaningful goals (61). Although children’s visits cannot improve their self-rated health, frequent visits by children can make older adults feel the concern of their family members, and children’s visits can play a role in monitoring the service quality of nursing homes, thus reducing depression and improving the psychological health of older adults. Our conclusions are consistent with many existing studies. For example, an early study in the United States concluded that social support from adult children improves the psychological well-being of older parents (62). A study in Japan also concluded that social support provided by children was significantly associated with mental health outcomes (63).

### 5.2 Recommendations

Based on the results of our study, we make the following recommendations. First, advancing age often means an increase in chronic diseases and the need for care. In the future, as China’s aging process deepens, more and more older adults will need to be admitted to institutions, and society should establish a sound social service system for older adults. A long-term care insurance system should be established as soon as possible to provide a stable source of funding for the development of nursing homes. At the same time, we should vigorously improve the service quality of nursing homes. By improving the service quality of nursing homes, we will gradually change people’s perceptions of poor service quality and create an environment where people are willing to live.

Second, the health situation of older persons living alone should be improved. Children should have a more open and tolerant attitude toward the remarriage of their older adult parents. It is also important to promote the creation of social capital at the community level. Some scholars have argued that promoting community activities may be an effective community intervention to promote mental health (64). Therefore, we should consider building more activity venues at the community level and organizing activities such as playing cards and chess, so that older adults living alone can have more opportunities to increase the frequency of their social activities.

Third, older adults living in nursing homes are generally physically weak (65), some seniors even feel abandoned by their children (66). Children should visit their parents residing in nursing homes regularly to enable their parents to feel the love and care of their children and to alleviate their state of depression. Our recommendations are consistent with the findings of existing studies, with one meta-analysis confirming that home visits help to alleviate depression in nursing home residents (67).

### 5.3 Limitation

To the best of our knowledge, there are few articles based on Chinese data that examine the effects of nursing home admission on health, and even fewer that focus on the role of social capital in buffering the negative impacts of living alone and living in a nursing home, an area in which this paper makes a marginal contribution. Several things could be improved in this study. First, due to the use of cross-sectional data, it is impossible to conclude the causal relationship between variables. Second, there were 15,874 respondents in the original database, but only 9,669 were finally included in our analysis, and a considerable number of respondents were excluded from the study because of missing values on some variables, which may have led to a biased distribution of the sample. Third, the CLHLS survey used nearly identical questionnaires for older adults in different living arrangements; for example, older adults living in nursing homes were not asked about their living situation in the nursing homes, which prevented us from conducting a more targeted analysis. Future research should explore the longitudinal dynamics and other forms of social capital in greater depth to further uncover the protective role of social capital and refine policy interventions to enhance the well-being of older adults.

## 6 Conclusion

Empirical evidence indicates that older adults living in non-family living arrangements are vulnerable to adverse physical and mental health outcomes, and that living alone and in nursing homes are significantly associated with depressive symptoms in older adults. More importantly, social capital was found to be a protective buffer against the negative effects of non-family living arrangements. To improve the health status of older persons, policymakers should develop policy measures to increase the family involvement of older persons living in nursing homes and organize more activities at the community level to encourage older adults living alone to become more involved in social activities.

## Data Availability

Publicly available datasets were analyzed in this study. This data can be found at: https://opendata.pku.edu.cn/dataset.xhtml?persistentId=doi:10.18170/DVN/WBO7LK&version=2.0.
